# mHealth for image-based diagnostics of acute burns in resource-poor settings: studies on the role of experts and the accuracy of their assessments

**DOI:** 10.1080/16549716.2020.1802951

**Published:** 2020-08-20

**Authors:** Lisa Blom

**Affiliations:** Department of Global Public Health, Karolinska Institutet, Stockholm, Sweden

**Keywords:** Mobile health, smartphones, injuries, remote consultation, specialists, gender differences, display quality, diagnostic accuracy, congruence model, positioning theory

## Abstract

Diagnostic assistance using mobile technology is instrumental to timely and adequate care in resource-scarce settings, particularly for acute burns. Little is known, however, as regards to how remote diagnostic consultation in burns affects the work process. This article reviews a doctoral thesis on this topic based on four studies conducted in the Western Cape, South Africa prior to and in a very early phase of the implementation of an app for burn remote diagnostic assistance. The aim was to increase knowledge on how remote diagnostic assistance for burn injuries can influence the role and work of medical experts in a resource-poor setting. The congruence model was used as a reference framework to study the ‘input’ (study 1), ‘tasks’ (studies 2 and 3) and ‘people’ (study 4) involved. The results show higher burn incidence in young children (75.4 per 10 000) and gender differences primarily among adults. The quality of images was considered by experts as better when viewed on smartphones and tablets than on computers. The accuracy of burn size assessments was high overall but low for burn depth (ICC = 0.82 and 0.53 respectively). Experts described four positions pertaining to remote consultations: clinical specialist, gatekeeper, mentor and educator. They perceived images as improving accuracy of consultation and stressed the need for verbal communication among clinicians during critical situations. In conclusion, experts are satisfied with the quality of images seen on handheld devices and can accurately assess burn size using these, yet burn depth assessment is more challenging without additional clinical information. mHealth for diagnostic assistance can benefit current image-based consultation by systematising information quality, introducing enhanced security and improved access to experts. Remaining challenges include the necessity of verbal communication in some instances and replacing existing informal organisational practices.

## Background

### Diagnostic support from clinical experts through mHealth

The provision of timely and adequate emergency care can reduce the global burden of disease, including injury morbidity and fatality [1–3]. A late initiation of patient care in resource-poor settings has many different causes [4–6]. One of them is the scarcity of specialists [4,6], which takes a toll on patient triage [6] and hinders informed decision-making among less experienced front line care providers [6].

The rapid development and widespread adoption of mobile technology have expanded the outreach of specialists by facilitating consultation, with a particular benefit for specialties like burns where images are instrumental to diagnosis [7]. At time of writing, in South Africa for example, considerable changes have and are taking place in healthcare where clinicians from different parts of the country have adopted mHealth apps to deal with triage of acute burns, either by using WhatsApp [8,9] or through formal channels with dedicated apps [10,11]. While this has led to fewer admissions [8,9] and changes in management plans [8], evidence regarding whether the displays of mobile devices provide sufficient quality for image-based consultations was, at the time of preparing and conducting the studies, scarce [12]. Burn assessments made by experts based on either conventional photographs [13,14], video [15] or computer screens [16] were known to be accurate but evidence was limited as regards handheld devices except for one study on an early generation of smartphone [17]. Yet, evidence from specialties like dermatology [18], radiology [19] and pathology [20] were quite encouraging in that regard. Furthermore, little is known as to how the work of experts is affected by these systems of consultations that bring along new working practices and conditions [21–23]. The perspective of specialists, often the initiators of, and definitely key actors in, these systems, have been documented in studies on system performance, once in place [24], but seldom, pre-implementation, as regards how they expect the work to become and thereby inform the implementation process. An investigation of the like, framed in an organisational theory, could be instrumental to both the development and implementation process of an app.

The four studies [25–28] of the thesis [29] took place when an app for acute burns was under development, intended to be used between emergency care nurses and physicians and burns experts in Western Cape province, South Africa [10,11]. The app was based on local initiatives and went through a long process of development in close collaboration with local stakeholders from burns and emergency medicine. The app was integrated into the Vula platform (www.vulamobile.com) in 2016 [10] and is since then available for free download to both Android and iOS devices by registered South African healthcare providers.

### Aim and research questions

The aim of the thesis was to increase the knowledge about how remote diagnostic assistance for burn injuries can influence the role and work of clinicians, with a specific focus on medical experts and the context of resource-poor settings. The following research questions were addressed:

- What are the distribution and circumstances of burn injuries treated in emergency services in resource-poor areas? Are there gender-related differences? (Study I)
- How do medical experts assess the image quality of clinical images viewed through handheld devices (smartphones and tablets) compared to when viewed on a laptop monitor? Is the assessment influenced by the clinical background of the participants? (Study II)
- How accurate is the image-based remote diagnosis of burns commonly presenting to emergency services in the Western Cape? Are remote assessments of comparable accuracy when made on handheld devices compared to on a computer? (Study III)
- How do experts perceive the opportunities in, and challenges of, the diagnostic and decision support system (an mHealth app for burns) about to be implemented? What does it imply for their role in the management of burn injuries? (Study IV)

### Enhanced understanding through the congruence model

**Figure 1.**
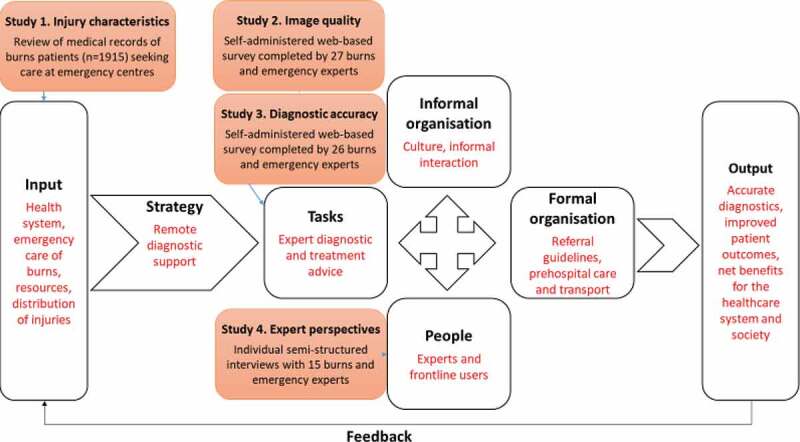
An application of the context of emergency care of burns (text in red) in light of the congruence model (adapted from [30]) and the four studies focusing on the work of experts.

The congruence model by Nadler and Tushman [30], with origin in organisational theory, was used to support the understanding of this coming change in healthcare. According to the congruence model, an organisation is a system with both social, structural and technical dimensions that consists of four key components: task, people, and formal and informal organisation. The model has been applied previously in the context of organisational change and healthcare implementation [31]. The organisation was, for the thesis, considered the organisation around burns care in the province and the focus was set on the changes in the work of experts that might follow the addition of images to remote diagnostic support (Figure 1). With this focus in mind three components of the model – input, tasks and people – were studied since those relate most closely to the work of experts when performing a diagnosis.

Input includes conditions in the environment, both external and internal such as regulations and historical context, influencing the specific organisation, e.g. available resources and the distribution of burns. The first study [25] feeds to the input and focused on the injury distribution. The strategy, herein considered to be remote diagnostic, triggers a transformation process in the four components of the organisation.

The tasks are the basic activities that should be done by the organisation which could be studied for example by investigating the nature of the tasks and the skills needed to perform the tasks. The second [26] and third [27] study examined image quality and diagnostic accuracy in relation to assessment of burns using smartphone or tablet.

The people perform the tasks, in this context they are the staff working in emergency care and the experts. Examining their knowledge, skills, needs and expectations is one way to study this component. The perspectives of experts on remote diagnostics were explored in the fourth study [32].

The remaining components of the model – formal organisation, informal organisation and output – were not studied in the thesis. High congruence between the four key components embed for high performance and good output [30] but it was not possible to assess level of congruence since not all components were examined.

## Methods

### Setting

The studies were conducted in South Africa, an upper-middle-income country [33] with extreme income inequality levels [34]. The implementation of the mHealth system was about to take place in Western Cape province where a high share of the population (19%) lives in informal settlements [35]. The strong association of burns with living conditions and economic deprivation creates a setting for high risk of burns [36,37]: unemployment can in some places reach 45% [38] and paraffin is the most common source of energy used for heating [38,39], but also locally for cooking and lighting [39].

The healthcare system of South Africa is two-tiered with private and public providers serving 16% and 84% of the population respectively [40]. In terms of public healthcare, the province’s 450 primary healthcare facilities and 34 district hospitals are assigned to take care of minor burns while the 8 regional hospitals care for minor and moderate burns. The province has one dedicated burns unit for children 0–12 years and another for adults 13 years and older which are expected to treat major and complex burns meeting the Western Cape provincial referral criteria [41,42].

### Study participants, data collection and data analysis

#### Study I. Injury characteristics

Study 1 [25] is a cross-sectional study based on case reports of patients who sought care for a burn injury at eight 24-h emergency centres, located in six hospitals and two community health centres, between 1 June 2012 and 31 May 2013.

The two community health centres and one of the hospitals are situated in suburbs to Cape Town predominated by informal settlements. The other five hospitals are found between 113 and 185 km from Cape Town in areas of a more rural characteristic. A standardised case report form based on WHO guidelines [43] was used. A total of 1915 patient cases were included for the final analysis.

Analysis took place in two different manners:

- **Gender differences**. Age-specific incidence rates for men and women were compiled and incidence rate ratios were used to examine gender differences in incidence. Two-sample tests of proportions were used to examine gender differences in burns circumstances, severity, length of stay and disposition of the patients.
- **Typical injury cases at emergency centres**. The data were first divided into children (0–12 years; n = 1 013) and adults (13 years and older; n = 900) and women (n = 904) and men (n = 1 011). The most common burns were thereafter ranked in each group separately in terms of body parts, either affecting a single or several body parts.

### Study II. image quality

In the second study [26], an online questionnaire was used including 18 images (clinical and non-clinical) viewed on three different devices (smartphone, tablet and computer) by 27 medical experts.

The questionnaire was developed in SurveyMonkey and the participants were asked to use a 7-point Likert scale (from 1 = terrible to 7 = excellent) to rate the quality of each image. Each survey ended with a few additional questions related to image quality and whether they had used the zoom function, experience of using the specific device for personal and professional purposes and how comfortable they would feel using the specific device for image-based remote consultation. Demographic data of the participants were captured at the end of the final survey.

Purposive sampling was used to identify physicians that were likely to act as consultants on acute burns, in addition to report having normal visual acuity and colour vision. Recruitment took place at two experts’ meetings in Cape Town in April 2015 and resulted in 27 participants – four South African burn surgeons and 23 emergency medicine specialists from sub-Saharan Africa and the USA. The participants repeated the same survey on each of the three devices while seated in the same spot with consistent lighting. The order of the devices was predetermined by six possible permutations and devices were wrapped in black paper to conceal the brand and model.

Linear regression clustered by participant was used comparing the quality ratings for the tablet and smartphone with those for the computer, stratified by image type.

### Study III. diagnostic accuracy

In the third study [27], 26 burns and emergency medicine experts assessed 51 images of burns using their own smartphone or tablet.

Images were selected to reflect the typical cases of burns in children and adults treated at emergency centres in the province identified in study I. A total of 5–6 images for each case were included with variation in terms of burn size, depth and mechanism.

The online questionnaire, built in SurveyMonkey, started with a few background questions, followed by the burns images (presented in random order) accompanied by basic patient information (age, sex and burn mechanism) and to which the participants were asked to estimate burn depth and size. The questionnaire ended with questions on their perceptions around the use of images in burns teleconsultation.

Purposive sampling was used to select participants with skills to diagnose burns, either by training or clinical practice. They were recruited from three groups: 1) tele-experts in the mHealth project for burns; 2) Swedish burns specialists involved in similar studies; 3) network of the burns specialists from group 1. The South African participants consisted of eleven emergency medicine specialists and eight burns specialists, while seven burns specialists from Sweden participated.

Participants were sent a link to the questionnaire and were instructed to complete it using their own smartphone or tablet. Eight participants repeated the questionnaire on a dedicated laptop computer two weeks or longer after completion of the first survey.

Diagnostic accuracy of assessments of burn size and depth was assessed using a two-way mixed effect intraclass correlation coefficient (ICC) with 95% confidence interval (CI) with bedside diagnosis as standard. All analyses were made on all images aggregated and stratified by children and adults. The ICC results were interpreted following these definitions [44]: <0.70 = low; 0.70–0.80 = acceptable; >0.80 = high.

### Study IV. expert interviews

The fourth study [32] was qualitative and based on semi-structured interviews with 15 medical experts prior to the implementation of the mHealth app for burns.

The study was informed by the information ecology framework by Nardi and O’Day, focusing on the human activities around a technology [45]. This framework, together with knowledge from the literature on user perspectives of telemedicine and local knowledge from the healthcare system supported the development of the interview guide. The guide started with some background questions on participants’ work and experiences of diagnosing burns bedside, current practices of diagnosing remotely and continued with questions on their experiences of apps and expectations of the mHealth app for burns that was about to be implemented.

Purposive sampling was used to select a broad range of participants that could all act as potential tele-experts for burns. Three groups were therefore considered: 1) the burns and emergency medicine experts enlisted for the mHealth project; 2) senior-level registrars in burns and emergency medicine specialty; 3) burns expert from another province for an outsider view. We recruited a total of fifteen participants, of which seven burns and eight emergency medicine specialists; seven female and eight males; and ten with prior knowledge of the app they would be interviewed about. The interviews were conducted by two interviewers and recorded electronically; they lasted 44–95 minutes.

The analysis was performed in two stages. Firstly with a data-driven thematic analysis [46] starting with open coding of seven full-length transcripts. Categories of types of interaction were thereafter formed after discussion within the research team and the remaining transcripts were coded. It was found that the interactions between experts and staff at POC were influenced by both clinical assessments and social circumstances why a theory to frame those multiple tasks and positions was searched for. Roles of medical experts have been discussed earlier [47] although with a perception of them being relatively stable over time despite the transforming environment in which physicians work. The positioning theory [48], developed by Harré and Van Langenhove, has been used in healthcare research previously [49,50], and provides a vigorous and interchangeable alternative to describe how individuals position themselves in interaction with others. The material was reviewed once more using the positioning theory as theoretical frame, comparing and contrasting the positions until four positions were developed. The use of the theory helped to clarify the changes in interaction that the implementation of the app would bring along compared to how remote consultation practices were taking place at the time of the interviews.

## Results

### What kind of injuries are seen at the emergency centres?

The majority of patients were 19 years and younger (60%) and two-thirds of those were under the age of five. Both children and adults were most commonly burned by hot liquids (69.4% and 60.3% respectively), followed by contact burns in children (12.6%) and fire burns in adults (17.6%) (unpublished results). Reports of interpersonal violence were found in 9.3% of burns cases. The burns were most often of minor or moderate severity (80.4%), and most patients were treated as outpatients (73.9%) and stayed shorter than 5 hours (65.1%). Nine in ten patients (88.8%) staying longer than 5 hours at the emergency centres met at least one criterion [41] for transfer to a burns centre (unpublished results).

***a) Gender differences***

All gender differences presented in this section are statistically significant and further details are found in the original article [25]. The youngest children (age 0–4 years) had the highest incidence rates (75.4 per 10 000) but gender differences were mainly found among adults. Men in the age groups 20–39 and over-55s had a higher incidence than women in fire burns (M/W RR 1.55; CI 1.00–2.42) and hot liquids burns (M/W RR 3.38; CI 1.59–7.82) respectively. Gender differences among adults were also found in circumstances where burn-injured men had a higher proportion compared to women that sought care during weekends (53.4% vs 44.4%), where alcohol use was suspected (7.6% vs 3.0%) and with reports of interpersonal violence (22.5% vs 15.6%). Moreover, adult men were transferred to higher levels of care more often (9.8% vs 5.2%) while a higher proportion of women were treated and discharged (82.8% vs 73.4%) despite similar severity.

***b) Typical injury cases at emergency centres***

The three most common single body parts burned in all four groups were lower extremities, trunk and upper extremities (Table 1). These were followed by hand and head burns among children, women and men while the fourth and fifth most common burns in adults were upper extremities and trunk combined and burns on the head.

**Table 1.** Five most common body parts burned among children and adults and women and men presenting to emergency centres in the Western Cape*.

Body part	Children (n = 1013) %	Adults (n = 900) %	Women (n = 904) %	Men (n = 1011) %
Lower extremities	13.1	13.9	15.8	11.4
Trunk including buttocks	12.1	11.3	12.1	11.6
Upper extremities excluding hands	9.6	9.9	10.1	9.4
Hands	8.6		7.0	7.5
Upper extremities and trunk		7.7		
Head	7.9	7.1	8.1	7.1

*Age was unknown for two patients

### Are the images of satisfactory quality and are the assessments made on handheld devices by medical experts accurate?

Quality ratings were significantly higher for images viewed on smartphones and tablets compared to when viewed on computer. The ratings were not affected by the medical specialty of the participants.

The assessments of burn size made on handheld devices of images portraying the burns most commonly seen at emergency centres (Table 1), were of high accuracy for both child (0.81; 95% CI 0.78–0.83) and adult (0.81; 95% CI 0.78–0.84) cases (Table 2). The assessments of burn size made of burns specialists were slightly more accurate than those made of emergency medicine specialists.

**Table 2.** Diagnostic accuracy of burn size and depth assessments made on handheld devices by all participants and by participant group.

Cases	Participants	Size	Depth
		ICC (95% CI)	ICC (95% CI)
Overall	All	0.82 (0.81–0.84)	0.53 (0.49–0.57)
	South African EM specialists	0.80 (0.77–0.83)	0.49 (0.43–0.55)
	South African burns specialists	0.87 (0.85–0.89)	0.64 (0.58–0.69)
	Swedish burns specialists	0.87 (0.84–0.89)	0.51 (0.43–0.59)
Children	All	0.81 (0.78–0.83)	0.61 (0.55–0.65)
	South African EM specialists	0.77 (0.72–0.81)	0.54 (0.45–0.62)
	South African burns specialists	0.90 (0.87–0.92)	0.75 (0.68–0.80)
	Swedish burns specialists	0.83 (0.78–0.87)	0.59 (0.48–0.68)
Adults	All	0.81 (0.78–0.84)	0.46 (0.40–0.52)
	South African EM specialists	0.79 (0.75–0.83)	0.44 (0.35–0.53)
	South African burns specialists	0.85 (0.81–0.89)	0.54 (0.43–0.63)
	Swedish burns specialists	0.87 (0.83–0.90)	0.45 (0.33–0.56)

The assessments of burn depth were of low accuracy overall but somewhat more accurate for child cases (0.61; 95% CI 0.55–0.65) than those of adult cases (0.46; 95% CI 0.40–0.52) (Table 2). South African burns specialists were slightly more accurate in their assessments of burn depth in child cases than those made of emergency medicine specialists.

The assessments made on computer followed the same pattern as those made on handheld devices with high accuracy of assessments of burn size (0.85; 95% CI 0.82–0.88) while those of burn depth were low (0.48; 95% CI 0.41–0.55).

### What do the experts foresee?

The interviews were conducted before the mHealth app had been implemented but at a time when the practice of using images for remote advice had already begun, mostly via WhatsApp as described by the experts, and the process of consulting were therefore already in a changing state. Experts described several different positions in regard to their practice of remote consultation; two related to the diagnosis and their clinical competence as clinical specialist and gatekeeper, and two to relations with other clinicians as mentor and educator.

#### Clinical specialist

This position entails both authority and responsibility and the clinical expertise in patient diagnosis and management. The process of making a remote diagnosis was perceived to be facilitated by the use of images:

It’s eyes on. I’m a surgeon, I need to see, even better, I need to feel, I need to be there, but if I can’t actually physically, I need to see because if you are not experiencing burns, what you are telling me, I can’t be sure that what I understand you to mean is actually what you mean. (BS.7)

Information of importance for diagnosis, for example the injury circumstances were still communicated verbally even when WhatsApp was used, and the experts expected that verbal communication would remain important even when using the new app. The dedicated system that the app offers was expected to make it easier to reach the experts compared to using the hospital switchboard or WhatsApp by systematically providing a link to the one expert on call, in particular when clinicians were not already connected through the contact lists on each other’s smartphones. Images as addition to the consultation, either via WhatsApp or the app, were perceived to improve the quality of remote advice to the extent that it is approaching that of a bedside diagnosis. This was described to create ambiguities about the division of patient responsibility between POC staff and remote consultants and those ambiguities were expected to persist even with the app in place.

#### Gatekeeper

The gatekeeper position involves ensuring an adequate patient flow based on patients’ needs. There was an overlap between the gatekeeper and the clinical specialist since the starting point of deciding on where patients should be managed relies on an accurate diagnosis. Experts emphasised the usefulness of including images in the referral process when referral needs were uncertain. Images were described to speed up the referral process by ensuring that the information provided to them by staff at POC was correct:

We’ll use WhatsApp, saying “This is what I’ve got, what do you think?” and we’ll make a decision on transfer from those pictures. And they can also e-mail us; then ultimately we use a formal e-mail reference. But for that initial decision it’s all made on cell phone images. (BS.4)

The app was expected to enhance the security of transmitting images compared to WhatsApp. The participants envisioned the app as a formalisation of the WhatsApp practice that would safeguard inclusion of relevant information:

To have an app, it just makes it more structured. The app asks you the right questions, so you give the right answers ….That’s really helpful because like I said, people don’t always know what’s important (BS.7)

The experts pointed out that verbal communication was often preferred for information of importance regarding the decision of patient transfer e.g. relating to patients’ social circumstances or staff capacity at POC. They foresaw that this information could risk being missed with the use of the new app. Furthermore, existing problems with lack of patient beds were described as potential barriers to POC staff’s willingness to use the app if the advices they received from experts could not be followed.

#### Mentor

This position involves the important task of experts of providing support to junior doctors and staff at POC. The experts perceived that management of burns patients, often in extreme pain, challenge staff at POC emotionally. The support of experts to POC was particularly pronounced when patient survival was uncertain:

Being overwhelmed by this really bad burn and knowing that it’s wrong for them to take up a bed in some way and having to make that decision, particularly for a junior provider to say well, I’m not going to do anything for this patient, I’m going to leave them here to die, or I’m going to let their family come in and then I’m going to intubate them and its … that actually needs quite a lot of support. (EM.8)

In these situations images were considered helpful to allow crucial management decisions to be based on accurate diagnoses:

I will usually ask them to send me a photo so I can just see what they are seeing, to just see if they are giving me an accurate description of the burn depth, because the worst thing is that you don’t want to make an error when it comes to withdrawal of therapy … (BS.3)

Although the experts suggested that the app could contribute to improved accuracy of diagnosis and supply information regarding palliative care, the importance of verbal communication in these situations was stressed by the experts and the mentor position is thus expected to be least affected by the implementation of the app.

#### Educator

Educating and empowering staff at POC was perceived by the experts as a core task in their work. Experts described how the use of images in remote consultation, sent via WhatsApp, had facilitated educational activities towards POC staff based remotely. The app was expected to provide additional educational benefits by the structured way of filling in information:

So I think the app will offer a more structured approach and the doctor also will learn about the information he needs to gain. Because the app will guide you: How was the patient burnt? How old is the patient? The structured approach … helps the junior doctor to form processes. (BS.6)

The experts speculated that this could potentially take over some of the experts’ educational tasks although a pleasant voice was considered important even in these situations.

## Discussion

The studies of the thesis have provided knowledge on three of the aspects of the congruence model – input, task and people – it is around these aspects that the main findings will be discussed.

### Main findings

#### Input – the acute burns treated in emergency centres

A few assumptions on the applicability of image-based consultation for burns could be drawn from examining the distribution and management of the injuries seen in emergency care. To start with, burns are common in this setting and the type of burns seen at emergency centres are by and large similar to what has been presented previously in resource-poor settings: young children are overrepresented among non-fatal burns [51–54] and the burns are mostly of minor and moderate severity [51–53], except for a few reports of more severe burns among children [55] and adults [51]. Most burns are due to hot liquids and this is in line with earlier studies concerning children [51–53,55,56] but contrasts previous studies on adults where flame burns often are more common [36,51,57]. Hence, no specific challenges are indicated by the kind of injuries presenting to emergency care – the input – for the tasks related to remote diagnostic assistance. One possible exception could be the larger burden of hot liquid burns among adults since scald burns could potentially pose additional challenges for remote assessments compared to flame burns [14]. In addition, the large share of the patients in emergency care in need of a discussion regarding referral to specialised care highlights the utility of consulting remotely supported by images. Based on these findings around the input, there seems to be a good potential for remote consultation in this setting. While the app was expected to improve the patient flow where patients are treated at the right level of care, certain structural problems related to the input were considered to be out of reach of what the app could solve, for example the problem of lack of patient beds.

The gender differences presented in study I are not easy to explain but similar results were found in a recent study from the area focused on paediatric burns [58], and the problem has also been discussed in earlier studies from the region [57,59]. It is unclear if these differences relate to actual gender differences in healthcare or to other factors, e.g. if there are certain severity factors that could not be captured in the material or individual patient requests such as if female patients more often demand to be cared for at the local hospital. Identifying the potential problem of gender-based differential treatment is a first step in preventing it from happening when using the app.

#### Task – performance of experts when assessing

The aspect of task is studied by examining the specific action of using handheld devices for burns assessments. Similar to findings using tablets for viewing radiological images [12], the experts perceive the quality of images viewed on smartphones and tablets as high, and are, as has been seen in other areas and regions [8,60], acquainted with using handheld devices for viewing images in clinical practice. The findings regarding accuracy are of a more conflicting nature. Burn size could be accurately assessed by experts by viewing images on handheld devices, similar to what has been shown in previous studies using photographs [13,14,17]. An accurate estimation of burn size is of particular importance for initial management decisions regarding e.g. resuscitation [61]. Contrary, assessments of burn depth are of lower accuracy and the results lands in-between those of earlier studies with more [14] and less [13] accurate remote assessments of burn depth. Even though assessments of burn depth is known to challenge experienced providers also at bedside [62], it is possible that these remote assessments could be made more accurate in clinical practice when more information surrounding the injury is included or when the experts could ask for additional information.

#### People – interactions among clinicians using smartphone messaging apps

The widespread practice of image-based consultation via WhatsApp reported by the experts and seen in the literature [8,9] indicates that there is both an interest and a place for digital solutions for burns care. The change in work practices that followed the initiation of using images and WhatsApp has altered all four positions of the experts by assisting in making an accurate diagnosis (clinical specialist), expediting referral decisions (gatekeeper), taking over responsibility for decisions (mentor) and expanding expertise (educator). Their acceptance of the new app might however be challenged by the high satisfaction expressed by many experts concerning WhatsApp and the benefits it has brought to consultation. Integration with some of the informal practices that are being created around the formal organisation around burns care could be problematic despite the anticipated additional benefits that the app could bring in terms of approachability of the experts (clinical specialist), security of transmission of patient information, relevance of information (gatekeeper) and educational benefits (educator). The networking feature in WhatsApp with profile pictures and group chat function is one example where the lines between the clinical and social worlds are blurred. Albeit appreciated by users, mixing social and personal discussions with those of clinical issues around patients raise both ethical and patient confidentiality concerns [63,64]. Indeed, some experts conveyed discomfort around these informal consultations but stated that the great benefits that images brought to remote consultations made it difficult to refuse using it since formal alternatives were not available at that time and the experts expressed a need for a formal system to ensure patient confidentiality and data security. Since the interviews were conducted, security of image transmission has improved with the end-to-end encryption of WhatsApp [60], but issues around storage of data persist [8], for example when clinical images are stored in the photo folder of clinicians’ private smartphones.

The difficulty in replacing verbal communication for certain situations was emphasised by the experts, in particular for critical issues related to the mentor position, and this has been reported previously [65]. Loss of context and personal contact can lead to misunderstandings when communicating via SMS on complex medical matters [66]. Verbal communication is part of the interaction taking place via WhatsApp, but it is not an available feature in the new app and thus could lower the interest in using it. Including verbal communication as a function in the app could, however, pose a problem for scaling up as it risks overburdening the pressured time schedules of experts.

#### Future research needs

The findings of the four studies highlight a number of research areas of particular value during implementation of the app. One is to follow up and deepen the understanding of the potential gender differences identified in referral and outpatient care, primarily to find out if they actually exist and, if so, explore the possible underlying reasons and address these. In addition, it is valuable to determine if these differences are maintained or reduced when referring through the app. Second is to study and monitor the diagnostic accuracy of remote consultations made through the app and whether the accuracy of those assessments varies with, for example, sex or mechanism and if use of the app affects the skills of staff at POC. Third, investigating the users’ perspectives of the app during use (usability under controlled conditions has been investigated by Klingberg et al. [67] but not in clinical practice) will support understanding of possible barriers and unexpected consequences in usage, and its effect on workflow and interactions with other providers.

### Methodological considerations

The studies of the thesis provide various perspectives on burns diagnostics deriving from data both quantitatively and qualitatively collected with three different approaches. Some prominent methodological considerations should be highlighted connected to the data collection approaches used. The first one relates to collection of hospital data (study I) that was done systematically over an extensive time period and with cross-checking to control consistency. Some underestimation of the burden of burns is expected since patients with less severe burns can seek care at other healthcare facilities in the area without being transferred to any of the included facilities. Also, gender differences might be depreciated since, at least for one of the hospitals, records of patients coming in after hours were lost to a high extent which is when male patients could be more likely to seek care. The problem with lack of information in the files is however not expected to affect women and men differently.

The second approach deals with data collected through questionnaires via online surveys (studies II & III) where images were presented in random order, all participants assessed all images with few missing answers, and they were both conducted in life-like lighting conditions. However, the few number of burns experts that exist impeded the possibilities for a random sample of participants although it is unclear if a random sample would have produced different results. Images were gathered from different sources and resolution could not be controlled which would also be the case for images sent in clinical practice. Whether this affected the quality perceptions or the ability of the participants to diagnose is uncertain. The accuracy results could be on the conservative side due to the limited information accompanying the images whereas, in clinical practice, the expert could request more information. The lower accuracy of the burn depth assessments could also partly stem from the inclusion of a few images with burns of several depths which has been reported previously [14].

Furthermore, the interview-based study (study IV) was strengthened by the heterogenous sample of experts including both women and men of various years of experience coming from different specialities and levels of care, as well as by the conduct of member checks for clarification and the fact that two interviewers with different backgrounds and perspectives were present during the interviews. Privacy concerns could arise since participants were selected from a small group of eligible individuals, but the response rate was high, and the participants generously shared both positive and negative experiences and expectations. Discussion and reflection within the research team were made throughout the study to highlight any potential emotions that could influence the process.

Lastly, using the congruence model as a conceptual framework for the thesis supported the understanding of a number of the aspects that challenge the implementation, acceptance and use of the new app. However, it was not possible to assess either the congruence of the organisation or the output since formal and informal organisations were not studied and the studies were conducted before the app was fully implemented and in use.

## Conclusions

The results of the thesis contribute important information concerning the introduction of an app for image-based consultation for acute burns in resource-poor settings.

To start with, the characteristics of patients treated in emergency care seem to be similar as those in other levels of care, with high proportions of children and hot liquid burns and burns of relatively minor or moderate severity. The fact that adult males are more at risk of sustaining burns than females is also relatively expected. However, the finding that men tend to receive more care than women do for seemingly similar injuries is surprising and the reasons behind this are unclear.

The potential for experts to use a smartphone for viewing images, nowadays a crucial component of remote consultation, is supported in two different ways. To begin with, experts perceive the quality of images as higher on smartphones and tablets than on computers. Moreover, they can assess burn size accurately by viewing images of burns on their smartphones. Additional clinical information might be needed to support image-based diagnosis of burn depth which could be provided in real clinical practice.

Lastly, four shifting positions are described by the experts working in remote consultation practices – clinical specialist, gatekeeper, mentor and educator – and the app is expected to change all of them, even when compared to current image-based consultation practices. The widespread use of WhatsApp in informal clinical communication point to a feasibility of the concept, although its popularity might intervene with the actual usage of the burn app. The app is expected to contribute additional benefits compared to current image-based consultation by improving quality of information, security issues and the ability to reach the experts. Remaining challenges include the necessity of verbal communication in some instances and replacing existing informal organisational practices.

## Paper context

Healthcare is changing with the wide adoption of mHealth apps but little is known about the accuracy of those new practices and how they affect work process and care delivery. This research focused on medical experts within the speciality of burns and presents several promising aspects in terms of feasibility and usefulness while also highlighting certain areas of concern related to informal communication practices and the value of verbal communication.
